# Valosin Containing Protein (VCP): A Multistep Regulator of Autophagy

**DOI:** 10.3390/ijms23041939

**Published:** 2022-02-09

**Authors:** Veronica Ferrari, Riccardo Cristofani, Barbara Tedesco, Valeria Crippa, Marta Chierichetti, Elena Casarotto, Marta Cozzi, Francesco Mina, Margherita Piccolella, Mariarita Galbiati, Paola Rusmini, Angelo Poletti

**Affiliations:** 1 Dipartimento di Scienze Farmacologiche e Biomolecolari, Università degli Studi di Milano, Via Balzaretti 9, 20133 Milan, Italy; veronica.ferrari@unimi.it (V.F.); riccardo.cristofani@unimi.it (R.C.); valeria.crippa@unimi.it (V.C.); marta.chierichetti@unimi.it (M.C.); elena.casarotto@unimi.it (E.C.); marta.cozzi@unimi.it (M.C.); francesco.mina@unimi.it (F.M.); margherita.piccolella@unimi.it (M.P.); rita.galbiati@unimi.it (M.G.); paola.rusmini@unimi.it (P.R.); 2 Unit of Medical Genetics and Neurogenetics, Fondazione IRCCS—Istituto Neurologico Carlo Besta, Via Celoria 11, 20133 Milan, Italy; barbara.tedesco@unimi.it

**Keywords:** VCP, autophagy, lysophagy, TFEB, TFE3, NF-κB, neurodegenerative disease

## Abstract

Valosin containing protein (VCP) has emerged as a central protein in the regulation of the protein quality control (PQC) system. *VCP* mutations are causative of multisystem proteinopathies, which include neurodegenerative diseases (NDs), and share various signs of altered proteostasis, mainly associated with autophagy malfunctioning. Autophagy is a complex multistep degradative system essential for the maintenance of cell viability, especially in post-mitotic cells as neurons and differentiated skeletal muscle cells. Interestingly, many studies concerning NDs have focused on autophagy impairment as a pathological mechanism or autophagy activity boosting to rescue the pathological phenotype. The role of VCP in autophagy has been widely debated, but recent findings have defined new mechanisms associated with VCP activity in the regulation of autophagy, showing that VCP is involved in different steps of this pathway. Here we will discuss the multiple activity of VCP in the autophagic pathway underlying its leading role either in physiological or pathological conditions. A better understanding of VCP complexes and mechanisms in regulating autophagy could define the altered mechanisms by which VCP directly or indirectly causes or modulates different human diseases and revealing possible new therapeutic approaches for NDs.

## 1. Introduction

Neurodegenerative diseases (NDs) are heterogeneous, frequently fatal and caused by the loss of neurons in different regions of the central or peripheral nervous system (CNS or PNS, respectively). NDs present very different phenotypes associated with the degeneration of a specific subset of neurons. Nevertheless, several NDs like Alzheimer’s disease (AD), Parkinson’s disease (PD), tauopathies, amyotrophic lateral sclerosis (ALS), spinal and bulbar muscular atrophy (SBMA), frontotemporal dementia (FTD), and Huntington’s disease (HD) share some clinical features and pathological mechanisms, like the presence of protein inclusions and protein quality control (PQC) system impairment. The presence of alterations in proteostasis leads to the inclusion of these NDs in the group of proteinopathies that also includes diseases known as multisystem proteinopathies and myopathies [1,2].

The PQC system is composed of chaperones and the degradative pathways, namely, the ubiquitin-proteasome system (UPS) and autophagy. Chaperones are proteins that, by cooperating with co-chaperones, are involved in the recognition of unfolded or misfolded proteins. Their role is based on supporting the proper folding of unfolded or misfolded proteins and, when this fails, on enhancing their clearance [3,4]. Valosin containing protein (VCP) is a chaperone-like protein, encoded by the *VCP* gene in humans, that has various roles in the PQC system being involved both in UPS and autophagy. VCP has a well-established role in enhancing misfolded protein degradation through the UPS, whereas VCP functions in autophagy are still not fully defined [5]. Here, we will present an overview of VCP focusing on its functions in regulating and supporting the autophagic flux and the impact that *VCP* disease mutations have on autophagy. The proper knowledge on VCP activity in this pathway could help in understanding the pathological mechanisms of VCP-related diseases, possibly opening new therapeutic approaches in proteinopathies.

## 2. VCP: A Master Regulator of the PQC System

**Figure 1:**
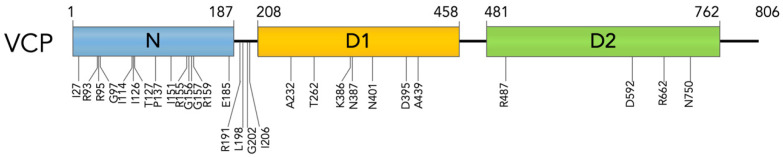
VCP mutants. Graphical representation of VCP structure and localization of mutated amino acids.

VCP (also known as Cdc48 in yeast and plants, CDC-48 in nematodes, and Ter94 in flies [6,7,8,9]) is an ATPase Associated with diverse cellular Activities (AAA^+^) that has a key role in maintaining cellular homeostasis. VCP is ubiquitously expressed in tissues [10]. At cellular level, VCP functions in many cellular compartments; VCP localizes mainly in the cytoplasm, while a smaller fraction binds to organelles or localizes in the nucleus, where it is implicated in different pathways [11,12,13,14,15]. VCP assembles and acts as a homo-hexamer, in which each monomer (represented in Figure 1) is structured in an N-terminal domain that interacts with adaptors and co-factors; in two ATPase domains, D1 and D2, that by hydrolyzing ATP concur, respectively, in the hexamer formation and in supporting VCP activity; and in a C-terminal domain that binds to a small subset of co-factors and adaptors cooperating with D2 activity [16,17,18,19] (see also review [20]). Generally, adaptor and co-factor motifs recognize the same binding site present on VCP; for this reason, the interaction with VCP is retained mutually exclusive. However, there are some co-factors/adaptors which are capable of binding simultaneously and collaborating in their activities as ubiquitin recognition factor in ER associated degradation 1 (UFD1)—Nuclear protein localization protein 4 (NPL4) complex [21,22]. The classification of the adaptors and co-factors is based on their interacting domain with VCP. The effectors that interact with VCP N-terminal domain present various recognizing motifs as: Ubiquitin regulatory X (UBX) motif present in UBX Domain Protein 1 (UBXN1) and UBX domain-containing protein 6 (UBXD1); VCP-interacting motif (VIM) present in small VCP interacting protein (SVIP); VCP binding motif (VBM) present in ataxin3; binding segment 1 motif (BS1, better known as SHP motif) present in both UFD1 and NPL4. The effectors that bind the C-terminal domain, present a PNGase/UBA (PUB) domain as Phospholipase A2 Activating Protein (PLAA) (reviewed in [23]).

The main role of VCP is to recognize and to extract ubiquitinated proteins from membranes, protein complexes, protein aggregates, or chromatin, modulating their ubiquitination or de-ubiquitination, and to enhance their degradation through the UPS or the autophagic pathway. The binding with different adaptors allows VCP to recruit ubiquitinated proteins in different cellular compartments. The binding with different co-factors modulates VCP activity by cooperating in recognizing and marking substrates, and it allows VCP to take part in many different cellular pathways, including ER-associated degradation (ERAD), organelle degradation, ribosome-associated degradation (RAD), regulation of autophagy, chaperone activity, chromatin-associated degradation, NF-κB activation, and membrane fusion [5,12,24,25,26,27,28,29]. Most of VCP activity is aimed at maintaining cellular proteostasis [30]. Indeed, *VCP* disease mutations are associated with signs of altered proteostasis in disease-affected tissues. Among VCP-associated diseases there are different NDs, including familiar forms of ALS, AD, and PD as well as a specific form of Charcot-Marie-Tooth disease (the CMT2Y). In addition, VCP is associated with 95% of the cases of multisystem proteinopathies (MSPs), as inclusion bodies myopathy Paget disease and frontotemporal dementia (IBMPFD) [31,32,33,34,35,36]. To date, more than 40 missense mutations in the *VCP* gene are associated with IBMPFD and almost 20 mutations are associated with ALS [37,38]. IBMPFD mutations are found at the interface between N-terminal and D1 domains whereas ALS missense mutations also localize in the D2 domain (R487H, D592B, R662C, N750S) as reported in Figure 1 and Table 1 [32,39,40]. *VCP* mutations do not lead to a complete loss of function; indeed, VCP-knockout mice are not vital [41]. Generally, VCP-patients, well resembled in VCP-mouse models, present disease onset only in the adulthood [42]. However, it was observed that *VCP* mutations alter binding to some co-factors, decreasing affinity or even preventing it [43,44], and decrease D1 affinity for ADP [45], leading to an increased ATPase activity of the D2 domain and a loss in the coordinated movement of N-terminal domain [45,46,47,48]. 

The most recurrent mutations are the missense mutations in Arginine-155 as R155C and R155H, which are both correlated to IBMPFD and ALS. Patients affected by these *VCP*-mutations present very heterogeneous phenotypes. However, R155C-patients generally present a more severe phenotype, with an earlier onset and a lower survival rate compared to R155H-patients [37]. Even though VCP-patients present different phenotypes, generally the affected tissue present similar alterations at cellular level. In skeletal muscles, fibers present inclusions positive to ubiquitin and VCP, rimmed vacuoles, and damaged lysosomes [35,89,90]. Similarly, affected neurons present nuclear and cytoplasmatic inclusions positive to VCP and ubiquitin [49]. Moreover, both muscles and neurons present TAR-DNA binding protein 43 (TDP-43, a nuclear DNA/RNA binding protein) mislocalization and aggregation. In fact, studies have demonstrated a correlation between VCP and TDP-43 cytoplasmatic redistribution, and TDP-43 cytotoxicity in the presence of VCP mutants [90]. These alterations can be correlated with PQC system impairment [35,89,90]. In particular, many studies associated these alterations with the malfunctioning of the autophagic pathway [91,92].

## 3. Autophagic Machinery

Autophagy is a highly conserved lysosomal degradative pathway, essential for the maintenance of cellular homeostasis. Autophagy is characterized by three main and distinct pathways that mediate the degradation of substrates in lysosomes. First is macroautophagy (comprising the chaperone-assisted selective autophagy, or CASA [93,94,95]); this can be non-selective or a highly regulated mechanism in which substrates, as protein aggregates or organelles, are recognized and engulfed in a double membrane organelle, the autophagosome. This organelle fuses with lysosome, allowing substrate degradation. Second is microautophagy, where lysosomes directly sequester fractions of cytoplasm and degrade them with acidic hydrolases. The selectivity of this mechanism is debated and reviewed in [96]. Third is chaperone-mediated autophagy (CMA), where only proteins with an exposed aminoacidic KFERQ sequence (or KFERQ-like) are exclusively recognized and subsequently internalized into lysosomes for degradation [97]. VCP is mainly found implicated in macroautophagy (hereafter autophagy).

**Figure 2:**
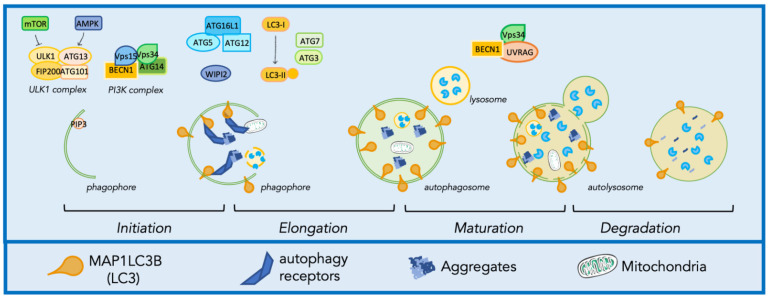
Molecular mechanism of autophagy. Autophagy involves a series of steps including initiation, elongation, maturation, and degradation. This figure was created using Servier Medical Art templates, which are licensed under a Creative Commons Attribution 3.0 Unported License; https://smart.servier.com (accessed 7 December 2021).

Autophagy includes a series of steps: initiation, elongation, maturation, and degradation, as described in Figure 2. During initiation, the nucleation of the autophagic membrane is induced by the formation of the preinitiation complex, composed by autophagy-related protein 13 (ATG13), autophagy-related protein 101 (ATG101), unc-51 like kinase 1/2 (ULK1/2), and FAK family-interacting protein of 200 kDa (FIP200). This complex is positively regulated by the AMP-activated protein kinase (AMPK) pathway and negatively regulated by the mammalian target of rapamycin (mTOR) pathway [98,99]. The preinitiation complex recruits to the ER the phosphoinositide 3 kinases (PI3K) complex, composed by ATG14, vacuolar protein sorting-associated proteins 34 and 15 (Vps34, Vps15), and Beclin1 (BECN1), which increases the production of the lipid phosphatidylinositol-3-phosphate (PtdIns3P) in the initial autophagic membrane, the phagophore [99,100]. PtdIns3P binding to the effector Wild-type p53-induced phosphatase1/2 (WIP1/2) mediates the recruitment of complexes involved in the activation of the phagophore elongation and expansion into the autophagosome. The complexes that coordinate the elongation are the ATG7-ATG3-Microtubule-associated proteins 1A/1B light chain 3B (MAP1LC3B) complex, which activates the lipidated form of MAP1LC3B, MAP1LC3B-II, and concurs for its binding to the membrane of the phagophore/autophagosome, and the ATG12-ATG5-ATG16L1 complex [101]. Once the autophagosome is formed, it fuses with a lysosome in a process called maturation, that is regulated by the Vps34/BECN1 and the UV radiation resistance associated (UVRAG) complex [102]. The fusion with lysosomes results in the degradation of substrates mediated by more than 60 hydrolytic enzymes contained in the lysosomal lumen maintained at a pH around ~4.5–5.0 [103]. As mentioned, autophagy substrates can have different natures, and their recognition and engulfment in autophagosomes are highly regulated. The regulation and the targeting of misfolded protein aggregates for disposal through autophagy is regulated by chaperone and co-chaperone complexes, as in the case of the CASA complex. This complex is composed of two molecules of the heat shock protein B8 (HSPB8), the Bcl2-associated athanogene 3 (BAG3), the heat shock protein 70 (HSP70) and the C-terminus of HSC70-interacting protein (CHIP). HSPB8 (together with BAG3) recognizes substrates while CHIP mediates their ubiquitination. HSP70 transiently interacts with BAG3 that binds the dynein motor complex, routing the substrate-CASA complex to the microtubule organization center (MTOC), where autophagosome nucleation occurs [104,105,106]. Here, substrates-conjugated ubiquitin (Ub) chains are recognized by autophagy receptors (AR) as Sequestosome 1 (SQSTM1/p62) or optineurin (OPTN) [107]. ARs mediate the recruitment of substrates to the phagophore by binding the components of the autophagic machinery localized on the forming membrane, such as MAP1LC3B-II, which is recognized thanks to the specific motif in AR sequence named LC3-interacting regions (LIR) [108].

In addition to these highly regulated post-translational modifications, the autophagic machinery is also regulated at the transcriptional level by specific transcription factors as transcription factors EB (TFEB) and E3 (TFE3). TFEB and TFE3 regulate the expression of genes encoding the proteins involved in lysosomal biogenesis and autophagy (coordinated lysosomal expression and regulation, CLEAR genes) [109,110]. TFEB and TFE3 are both phosphorylated and inactivated in cytoplasm by mTOR and are dephosphorylated and activated by calcineurin (PPP3) [111,112]. In specific stress conditions as heat stress, the transcription factor NF-κB is implicated in autophagy regulation. Indeed, under these conditions, NF-κB positively regulates the expression of genes encoding proteins that route substrates to autophagy such as HSPB8 and BAG3, involved in the previously described CASA, promoting the degradation of damaged proteins or other substrates, selectively routing them to autophagy [113].

Autophagy has an essential activity in the preservation of cellular homeostasis. The alteration in any of its steps or the malfunctioning of any of its various players are associated with a broad number of organ-specific or systemic diseases as well reviewed in [114] and in [115]. In particular, the proper functioning of autophagy machinery is crucial to maintain homeostasis in cells of tissues characterized by a very low number of mitotic cells, or of tissue exposed to stressful conditions like neurons and skeletal muscle. The combined presence of stressors and low cell division rate, which is an essential mechanism to clear dysfunctional cellular components, confers an increase in cell reliance on the efficient removal of toxic elements to ensure cellular homeostasis and to prevent cell death [116,117]. In NDs, mutation of genes can cause, on one hand, defects in degradation pathways, as mutations in SQSTM1/p62, VCP and OPTN encoding genes, with an accumulation of misfolded protein aggregates and, on the other hand, the overproduction of misfolded proteins as α-Synuclein, Huntingtin, and Superoxide dismutase 1, that may overwhelm degradative pathways [32,114,118,119,120,121,122,123]. In this context, the modulation of a regulator of the autophagic pathway could lead to an amelioration of the phenotype.

It must be emphasized that a tight regulated equilibrium exists between autophagy and the alternative intracellular degradative system, UPS, with the proteasome as a core component. The proteasome processes ubiquitinated substrates, but only in their monomeric and non-aggregated forms. Several factors contribute to maintains this equilibrium in cells as, for example, the routing system based on the interaction of HSP70/CHIP with HSPB8/BAG3 (described above for the CASA complex), or with BAG1, which specifically routes misfolded ubiquitinated substrates to the proteasome. The impairment of the proteasome leads to an upregulation of HSPB8 and BAG3, enhancing the clearance of undegraded UPS-substrates through autophagy. Meanwhile, alteration in any step of the autophagic pathway leads to an increase in BAG1 levels promoting degradation of unfolded protein through the UPS [124,125,126].

## 4. VCP Role in Autophagy

**Figure 3:**
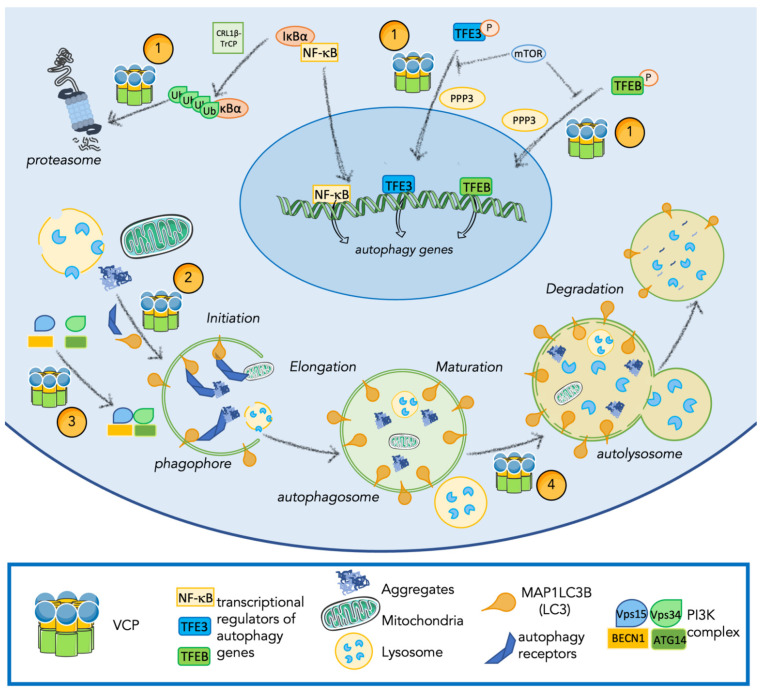
Overview of different roles which VCP plays in the autophagy pathway. VCP regulates autophagy and autophagic substrates degradation at different steps: (1) VCP is involved in regulating activation of transcription factors as NF-κB, TFE3, and TFEB; (2) VCP cooperates in the routing to autophagy of misfolded protein aggregates and organelles including lysosomes and mitochondria; (3) VCP enhances the formation of the PI3K complex promoting autophagy initiation; (4) VCP is involved in autophagosome maturation. This figure was created using Servier Medical Art templates, which are licensed under a Creative Commons Attribution 3.0 Unported License; https://smart.servier.com (accessed 7 December 2021).

Data on the role of VCP in autophagy are still controversial. Indeed, *VCP* mutations or silencing were associated both with the impairment of autophagy and with the accumulation of SQSTM1/p62 and MAP1LC3B-II suggesting the role of VCP in the activation and the support of autophagy [5,52]. However, some reports showed an opposite effect of VCP in autophagy in which the inhibition of VCP leads to SQSTM1/p62 degradation that is reverted by autophagy inhibition with Bafilomycin A, a late-step autophagy inhibitor supporting the inhibitory role of VCP in the autophagic pathway [127]. In addition, some VCP amino acid residue substitutions such as P137L and R93C were shown to stimulate autophagosome formation and its fusion with lysosomes [128]. More recent findings pointed out a complex relationship between VCP activity and autophagy regulation [5]. Indeed, it was discovered that VCP acts at different steps of the autophagic pathway as represented in Figure 3.

### 4.1. VCP Is Implicated in Transcriptional Regulation of Genes Involved in Autophagy and Inflammation

VCP has been found implicated in the regulation of diverse transcriptional factors that modulate the expression of autophagy-related genes [129,130]. In particular, in the presence of lysosomal damage, VCP regulates the activation of TFEB and TFE3 that promote the expression of the CLEAR genes [109,110]. When lysosomal damage is induced, the silencing or inhibition of VCP activity stabilizes TFEB activation [129]. Our unpublished data show that mutations in the gene encoding VCP lead to the specific activation of TFE3, suggesting the involvement of VCP also in such regulation of this autophagy-related transcription factor. The mechanism through which VCP regulates TFEB and TFE3 activation is still not clear, at present. It has been postulated that the regulation is based on a direct interaction with modulators of the transcription factors, or alternatively to an indirect mechanism associated with lysosome stability. Indeed, both TFEB and TFE3 when inactivated bind to lysosomes [131,132], and lysosomal alterations can promote their activation [133,134]. As will be described extensively below, VCP has a key role in lysosome stability, that could indirectly regulate TFEB and TFE3 activity.

VCP also modulates the activation of NF-κB, a transcription factor able to promote the expression of genes encoding cytokines [135]. In addition, as mentioned above, NF-κB regulates the expression of genes for HSPB8 and BAG3. These proteins bind HSP70 and CHIP forming the CASA complex which recognizes and marks substrates, mediating their degradation through autophagy [93,95,104,136]. NF-κB activation is triggered by plasma membrane receptors such as toll-like receptors or interleukin-1 receptors, which promote the downstream phosphorylation and K63-ubiquitination of proteins involved in the regulation of NF-κB activation and translocation to the nucleus [137]. Inactivated NF-κB localizes in the cytoplasm bound to IκBα. NF-κB activation requires the phosphorylation of both NF-κB and IκBα, then IκBα is ubiquitinated by the culling-RING ligase and β-transducin repeat containing protein (CRL1β-TrCP) complex. VCP is recruited by this complex to mediate IκBα proteasome degradation [138,139]. The VCP co-factors implicated in these pathways are not well characterized, but data suggested that co-factors p47 and Fas-associated factor 1 (FAF1) are able to inhibit the activation of NF-κB [140,141].

### 4.2. VCP Regulates Autophagy Initiation

Autophagy initiation is regulated by an increase of PtdIns3P in the phagophore membrane that recruits initiation complexes and membranes, permitting phagophore elongation and the formation of the autophagosome [99]. As mentioned above, the regulation of PtdIns3P production is mediated by the ATG14-Vps34-Vps15-BECN1 complex. Recently, it has been shown that VCP promotes the ATG14-Vps34-Vps15-BECN1 complex activity through two distinct mechanisms. First of all, VCP favors BECN1 deubiquitination, by binding its co-factor Ataxin3, a deubiquitinating enzyme, which frees BECN1 from ubiquitin chains. This prevents BECN1 degradation through the UPS and increases BECN1 cytoplasmic levels, favoring its binding in the complex. In parallel, VCP also binds ATG14, in a BECN1- and Ataxin3-independent manner, promoting complex assembly. Through these two mechanisms, VCP enhances the formation and the stabilization of ATG14-Vps34-Vps15-BECN1 complex resulting in an increase of PtdIns3P production. In addition, Hill and colleagues also speculated a possible role of VCP in the modulation of Ataxin3/MAP1LC3B binding. Indeed, the binding between Ataxin3 and MAP1LC3B was previously identified. Since VCP is able to bind to both proteins, it could mediate this interaction through a still unknown mechanism, influencing with a third pathway autophagic initiation [5,142]. Together these novel findings confer to VCP an important role in autophagy initiation.

### 4.3. VCP Routes Substrates to Autophagy

VCP chaperone-like activity has been proven in the presence of various protein aggregates, such as those formed by the amyloid Aβ-42 peptides characteristic for AD, or by mutant huntingtin (mHtt) characteristic for HD [143]. VCP activity on aggregates depends on the concentration of the protein prone to aggregate: VCP can either promote disaggregation or aggregation of misfolded proteins as both Aβ-42 peptides and mHtt [143,144]. VCP interaction with mHtt aggregates was well characterized: N-terminal VCP domain interacts with mHtt and promotes the degradation of its aggregates through autophagy. Nevertheless, VCP partners and mechanisms for degradation of mHtt aggregates are still not fully defined. Some studies showed that VCP directly interacts with histone deacetylase 6 (HDAC6), a modulator of autophagy that is involved in aggresome formation, suggesting that VCP cooperates with aggregates degradation through autophagy. Indeed, HDAC6 is needed to regulate the dynein-mediated transport of ubiquitinated substrates to the aggresome, promoting substrate degradation through autophagy [145,146,147,148]. In support of this, it was recently shown that VCP is required for both aggresome formation and clearance. Moreover, the VCP co-factors UBXN1 and UFD1-NPL4 complex were identified to co-localize with aggresomes and increase their binding with VCP in the presence of Bortezomib, a UPS inhibitor that enhances aggresome formation. However, neither UBXN1 nor UFD1-NPL4 complexes are essential for VCP activity in aggresome formation, suggesting the presence of other unidentified co-factors. Finally, it was demonstrated that also UBXN1 interacts with mHtt aggregates and is involved in their degradation [148]. In addition to the VCP role in aggresome formation and clearance, other data suggest that VCP can directly mediate substrate degradation through autophagy. Indeed, two LIR motifs are present in the N-terminal VCP domain and mediate the VCP binding to MAP1LC3B. It has been demonstrated that VCP-MAP1LC3B interaction mediates mHtt aggregates disposal by direct recruitment of VCP-bound substrates to MAP1LC3B-forming autophagosomes. In addition, the stabilization of VCP-MAP1LC3B binding using a specific inhibitor of D1 domain, increases mHtt aggregates [149,150].

In addition to misfolded protein aggregate degradation, VCP is implicated in disrupted organelle degradation, as mitochondria, through autophagy (mitophagy). The VCP role in mitophagy is quite well established. Damaged mitochondria prevent the degradation of proteins such as PTEN-induced kinase 1 (PINK1) stabilizing the exposure of the outer membrane, which promote the ubiquitination of substrates and the recruitment of E3-ligases amplifying the ubiquitination of proteins. VCP is recruited by the complex UFD1-NPL4 on the surface of the damaged mitochondrial membrane. Here, VCP binds to proteins as mitofusin, marked with K48-ubiquitinated chains. These proteins are substrates of VCP and are eliminated via UPS. The degradation of these proteins is necessary for mitochondria degradation, conferring to VCP an essential role in damaged mitochondria degradation [28,151]. In addition, it was shown that VCP localized to mitochondria recruits MAP1LC3B bound to the forming autophagosome, thus promoting mitochondria degradation. In HD models, VCP-MAP1LC3B mediated mitophagy results as toxic; indeed mHtt cause the accumulation of VCP on mitochondria membranes, triggering an overactivation of mitophagy [149].

Overall, VCP has a relevant role in routing substrates to autophagy, either by interacting with mediators that route substrates to autophagy or by the direct linking of the substrate to the forming autophagosome.

### 4.4. VCP Regulates Lysosome Stability and Degradation

The lysosome is the hub of the autophagy machinery. Its proper functionality is preserved and assured by complex regulatory mechanisms [152]. If lysosomes present alterations or damages that cannot be repaired, selective lysosomal degradation through autophagy (lysophagy) is promptly activated [153]. Lysophagy is highly regulated and consists in the marking of altered lysosomes and their disposal through both ubiquitin-dependent and independent mechanisms [152]. Damaged lysosomes expose β-galactosides that recruit galectin-3 (GAL-3) and -8 (GAL-8) and, consequently, ubiquitin ligases [27]. GAL-3 engages tripartite motif containing 16 (TRIM16), which in turn recruits additional E3 ligases and initiation factors of the autophagic machinery, such as ATG16L1 and ULK [154,155]. Ligases mark damaged lysosomes with K63-linked ubiquitin chains that are recognized by ARs, as SQSTM1/p62, linking damaged lysosomes to the forming phagophore [154,156]. In a subset of damaged lysosome, E2 enzymes, as ubiquitin conjugating enzyme E2 Q family like 1 (UBE2QL1), promote a K48-linked ubiquitin chain marking, that recruits VCP complexed with its co-factors PLAA, UBXD1, and ubiquitin thioesterase OTU1 (YOD1) [27,157]. The K48-ubiquitinated targets of VCP in lysosomes are still not identified. Nevertheless, VCP activity results essential for the degradation of this subset of damaged lysosomes. Indeed, VCP patients present in the sarcoplasm of skeletal muscle accumulation of GAL-3-positive vesicles which are also positive to caveolin-3 (Cav3). Cav-3 is a structural protein that in the presence of disease mutations was found accumulated on lysosomes and late endosomes [27,158]. Alterations in lysosomes were observed and well-recapitulated also in VCP-mouse models [27,129]. In VCP-mouse, conditional VCP knock-out in skeletal muscle leads to necrotic myopathy with increased autophagic protein levels and damaged lysosomes, whereas the knock-in of the disease mutant VCP, R155H, did not show an increase in autophagic markers, but presented accumulation of GAL-3 [129].

In addition to the role in lysophagy, the activity of VCP has been proven essential to maintain lysosomal stability. Studies on tubular muscle lysosomes show that VCP inhibition or downregulation causes fragmentation of lysosomes [159]. Moreover, the presence of VCP mutants R155H, R191Q and A232E is associated with disruption of lysosome morphology [159]. Recently, the VCP co-factor SVIP was identified as the mediator of the interaction between VCP and lysosome. Firstly, it has been proven that SVIP overexpression leads to a re-localization of VCP to lysosomes. In addition, it has been shown that SVIP-dependent VCP recruitment is essential to stabilize lysosomal structure and function. The downregulation of SVIP prevents VCP-lysosome interaction leading to alteration in lysosome stability, which is rescued only by a synthetic reconstitution of VCP-lysosomal binding. In support of SVIP essential cooperation in the VCP role, it was identified that the P134L amino acid residue substitution in VCP prevents its binding with SVIP and is causative of aggregate formation suggesting that P134L VCP inability to bind to SVIP and to target lysosomes could cause defects in lysosome functionality [160].

### 4.5. VCP Regulates Autophagosome Maturation

The evidence of the VCP role in regulating autophagic maturation is deduced by the accumulation of immature autophagosomes in the presence of *VCP* dominant-negative (DN) mutants or upon *VCP* gene silencing in cell and animal models [51]. VCP malfunctioning or silencing are correlated with an increase of MAP1LC3B-II and SQSTM1/p62 protein levels that do not occur if an inhibitor of a late step of autophagy is added. These are typical signs of autophagic blockage [161]. In addition, by observing autophagosome formation and fusion with lysosome by immunofluorescence analysis, it was demonstrated that the silencing of *VCP* or the overexpression of a DN mutant of *VCP* resulted in accumulation of autophagosomes with increased size, if compared with controls [51,52]. Nonetheless, the presence of VCP mutants related to diseases leads to an accumulation of autophagosomes fused with lysosomes. Myoblasts derived from VCP-patients are characterized by an accumulation of vacuoles positive to lysosome-associated membrane glycoprotein 1 (LAMP-1) and lysosome-associated membrane glycoprotein 2 (LAMP-2), which are lysosome markers. Thus, in these conditions autophagosome maturation seems impaired at a late stage, since autophagosomes accumulate after acidification and delivery of lysosomal hydrolases [51]. Despite these evident signs of alteration in the autophagic maturation, that suggests an involvement of VCP in this step of autophagy, to date, no clear mechanisms have been described.

## 5. Conclusions

VCP has multiple activities throughout the autophagic pathway. VCP is involved in different steps of the autophagic process as: autophagy activation by concurring in the regulation of the transcription factors NF-κB, TFEB and TFE3; autophagy initiation by promoting the formation and stabilization of ATG14-Vps34-Vps15-BECN1 complex resulting in increased PtdIns3P levels; and autophagy maturation by cooperating in autophagosome and lysosome fusion through a still unknown mechanism. In addition, the role of VCP is fundamental for lysosomal homeostasis and functionality. Indeed, VCP binds to lysosomes and, through SVIP-dependent mechanisms, preserves lysosome morphology and activity; in cooperation with PLAA and UBXD1, VCP regulates damaged lysosome degradation. Finally, VCP has a key role in enhancing the degradation of substrates, such as protein aggregates or organelles. VCP promotes clearance of substrates through different mechanisms: by cooperating in the formation of aggresomes, thanks to its co-factors UBXN1, UFD1 and NPL4, and in the clearance of aggresomes by interacting with HDAC6; by promoting substrate degradation via autophagy by direct binding with MAP1LC3B; and by enhancing the disposal of proteins which is essential to regulate organelle degradation. The VCP essential role in autophagy is also proven in VCP-related diseases. Indeed, *VCP* mutations have been associated with several signs of autophagy alterations, as the presence of ubiquitin inclusions and vacuoles accumulation. The exact mechanism of the malfunctioning of autophagy in the presence of *VCP* mutations is still greatly debated. However, as this review highlights, VCP multistep activities in autophagy suggest a presumable loss of functioning of VCP. Here, we discussed the known mechanisms that link VCP to autophagy, but most of these mechanisms need to be better defined. The identification of VCP effectors and adaptors that mediate VCP activity in each step of autophagy could define new targets to promote autophagy and the clearance of protein inclusions, a pathological feature of NDs. Therefore, a positive modulation of autophagy in NDs could ameliorate the phenotype of these diseases.

## Figures and Tables

**Table 1:** The table lists most *VCP* mutations. It reports the localization and the amino acid residue substitution in the protein encoded by each *VCP* mutation, the mutation at nucleotide level and the associated diseases (IBM, inclusion body myopathy; PDB, Paget’s disease of bone; FTD, frontotemporal dementia; PD, Parkinson disease; ALS, amyotrophic lateral sclerosis; CMT2Y, Charcot-Marie-Tooth disease type 2).

Domain	Protein Position	Amino Acid Residue Substitutions	Nucleotide Mutation	Associated Disease	References
*N domain*	I27	p. I27V	c.79A>G	IBM, FTD, PDB	[49,50,51]
	R93	p. R93C	c.277C>T	IBM, PDB, FTD	[52,53]
		p. R93H	278G>A	HSP	[54]
	R95	p. R95C	283C>T	IBM, ALS	[55]
		p. R95H	284G>A	AD	[33]
		p. R95G	283C>G	IBM, PDB, FTD, ALS	[35,56]
	G97	p. G97E	290G>A	IBM, PDB, FTD	[57,58]
	I114	p. I114V	340A>G	ALS	[59]
	I126	p. I126F	376A>T	IBM, PDB, FTD	[60]
	T127	p. T127A	379A>G	FTD, AD	[61]
	P137	p. P137L	410C>T	IBM, PDB, FTD	[62,63]
	I151	p. I151V	451A>G	IBM, ALS	[64,65]
	R155	p. R155S	463C>A	IBM, PDB, FTD	[62]
		p. R155L	464G>T	IBM, PDB, FTD	[66]
		p. R155H	464G>A	IBM, PDB, FTD, ALS	[35,53,62,67,68,69]
		p. R155C	463C>T	IBM, PDB, FTD, ALS	[35,52,69,70,71]
		p. R155P	464G>C	IBM, PDB, FTD	[35]
	G156	p. G156C	466G>C	ALS	[72]
		p. G156S	466G>A	IBM, PDB, FTD	[73]
	G157	p. G157R	469G>C/469G>A	IBM, PDB, FTD	[62,74]
	M158	p. M158V	472A>G	PDB, ALS	[75]
	R159	p. R159G	475C>G	ALS, FTD	[32]
		p. R159C	475C>T	IBM, FTD, PD, ALS	[34,36,69,76]
		p. R159H	476G>A	IBM, PDB, FTD, ALS	[59,62,77,78]
	E185	p. E185K	553C>T	CMT2Y	[31]
*N-D1 linker*	R191	p. R191G	571C>G	BM, ALS	[69]
		p. R191Q	572G>A	IBM, PDB, FTD, ALS	[32,35,56,62,69]
	L198	p. L198W	593T>G	IBM, PDB, FTD	[66,79]
	G202	p. G202W	604G>T	IBM, FTD	[80]
	I206	p. I206F	616A>T	IBM, PDB, FTD	[81]
*D1 domain*	A232	p. A232E	695C>A	IBM, PDB	[35,56]
	T262	p. T262A	784A>G	IBM, PDB, FTD	[82]
	K386	p. K386E	1158T>C	IBM	[83]
	N387	p. N387H	1159A>C	IBM, FTD	[79]
		p. N387S	1160A>G	IBM, PDB, FTD	[84]
		p. N387T	1160A>C	ALS	[39]
	D395	p. D395A	1184A>C	FTD	[85]
	N401	p. N401S	1202A>G	FTD, ALS	[61]
	A439	p. A439S	1315G>T	IBM, PDB	[62]
		p. A439P	1315G>C	IBM, PDB, FTD	[86,87]
		p. A439G	1316C>G	IBM, FTD	[80]
*D2 domain*	R487	p. R487H	1460G>A	FTD, ALS	[88]
	D592	p. D592N	1774G>A	ALS	[32]
	R662	p. R662C	1984C>T	ALS	[39]
	N750	p. N750S	2249A>G	ALS	[40]

## Data Availability

Not applicable.
